# Deciphering the neuroprotective mechanisms of RACK1 in cerebral ischemia‐reperfusion injury: Pioneering insights into mitochondrial autophagy and the PINK1/Parkin axis

**DOI:** 10.1111/cns.14836

**Published:** 2024-08-04

**Authors:** Lanqing Zhao, Yu Chen, Hongxi Li, Xiaoxu Ding, Jinwei Li

**Affiliations:** ^1^ Department of Sleep Medicine Center The Shengjing Affiliated Hospital, China Medical University Shenyang Liaoning People's Republic of China; ^2^ Department of Otorhinolaryngology Head and Neck Shengjing Hospital of China Medical University Shenyang Liaoning People's Republic of China; ^3^ Department of Pain Management Shengjing Hospital of China Medical University Shenyang Liaoning People's Republic of China; ^4^ Department of Neurology/Stroke Center The First Affiliated Hospital of China Medical University, China Medical University Shenyang Liaoning People's Republic of China

**Keywords:** cell apoptosis, cerebral ischemia‐reperfusion injury, mitochondrial autophagy, mitochondrial function, neuroprotection, PINK/Parkin pathway, RACK1

## Abstract

**Introduction:**

Cerebral ischemia‐reperfusion injury (CIRI) is a common and debilitating complication of cerebrovascular diseases such as stroke, characterized by mitochondrial dysfunction and cell apoptosis. Unraveling the molecular mechanisms behind these processes is essential for developing effective CIRI treatments. This study investigates the role of RACK1 (receptor for activated C kinase 1) in CIRI and its impact on mitochondrial autophagy.

**Methods:**

We utilized high‐throughput transcriptome sequencing and weighted gene co‐expression network analysis (WGCNA) to identify core genes associated with CIRI. In vitro experiments used human neuroblastoma SK‐N‐SH cells subjected to oxygen and glucose deprivation (OGD) to simulate ischemia, followed by reperfusion (OGD/R). RACK1 knockout cells were created using CRISPR/Cas9 technology, and cell viability, apoptosis, and mitochondrial function were assessed. In vivo experiments involved middle cerebral artery occlusion/reperfusion (MCAO/R) surgery in rats, evaluating neurological function and cell apoptosis.

**Results:**

Our findings revealed that RACK1 expression increases during CIRI and is protective by regulating mitochondrial autophagy through the PINK1/Parkin pathway. In vitro, RACK1 knockout exacerbated cell apoptosis, while overexpression of RACK1 reversed this process, enhancing mitochondrial function. In vivo, RACK1 overexpression reduced cerebral infarct volume and improved neurological deficits. The regulatory role of RACK1 depended on the PINK1/Parkin pathway, with RACK1 knockout inhibiting PINK1 and Parkin expression, while RACK1 overexpression restored them.

**Conclusion:**

This study demonstrates that RACK1 safeguards against neural damage in CIRI by promoting mitochondrial autophagy through the PINK1/Parkin pathway. These findings offer crucial insights into the regulation of mitochondrial autophagy and cell apoptosis by RACK1, providing a promising foundation for future CIRI treatments.

## Graphical abstract

This study reveals the key role of RACK1 in CIRI. The study finds that RACK1 regulates mitochondrial function and autophagy. The study confirms the centrality of the PINK1/Parkin pathway. The study offers new strategies for treating CIRI. The study provides comprehensive in vitro and in vivo evidence.

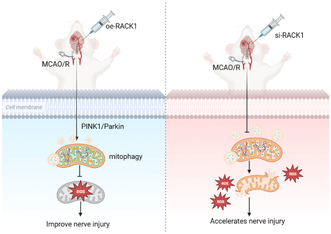

## 1 INTRODUCTION

Cerebral ischemic vascular disease ranks as the second leading cause of death in humans. Restoring blood supply to the ischemic area is crucial in treating such diseases; however, reperfusion often leads to secondary brain damage, clinically known as cerebral ischemia‐reperfusion injury (CIRI).^1^ The occurrence of CIRI can result in further damage to the neurocytes in the ischemic area and worsen patients' clinical outcomes.^2^ Yet, the pathological mechanism of CIRI remains incompletely understood at present.^3^ The global incidence of stroke and its concomitant complications, such as CIRI, are on the rise in tandem with the aging global population. These increasing occurrences add significant economic and psychological strain on healthcare infrastructures and the families of affected individuals.^4^

CIRI involves complex pathological processes, such as energy disruption, intracellular calcium homeostasis loss, and cellular acidosis.^5^ Recent studies have indicated a close association between CIRI and mitochondrial dysfunction.^6^ The impairment of mitochondrial function under ischemic conditions leads to an inadequate cellular energy supply, escalating cell death.^7^ Reperfusion exacerbates this by promoting the generation of oxygen free radicals, further damaging mitochondria and propelling cell apoptosis.^8^, ^9^

The role of mitophagy, the selective degradation of dysfunctional mitochondria, is receiving increasing focus in the study of CIRI.^10^ This process is critical for removing damaged mitochondria, maintaining cellular function, and proving particularly vital under stress conditions such as ischemia and hypoxia.^11^, ^12^ The PINK1/Parkin pathway is a core player in mitophagy, spearheading the elimination of damaged mitochondria.^13^

Nonetheless, the molecular intricacies of mitophagy in CIRI are not fully unraveled. RACK1 (receptor for activated C kinase 1) is a multifunctional protein widely distributed within cells, associated with numerous cellular signaling pathways.^14^, ^15^ Additionally, studies have indicated that RACK1 protects against secondary brain injury.^16^ Emerging evidence suggests a potential relationship between RACK1 and mitochondrial autophagy within the context of the PINK1/Parkin pathway.^14^, ^15^, ^17^ This study endeavors to intensively explore the functional role of RACK1 in CIRI and its regulatory influence on mitochondrial autophagy. Unveiling these molecular mechanisms can pave the way for identifying innovative targets for CIRI treatment, thereby enhancing patient outcomes and life quality.

In summary, an in‐depth analysis of the role of RACK1 in mitochondrial autophagy within CIRI could unravel novel molecular targets, significantly contributing to the development of advanced therapeutic approaches and positively impacting patient care and prognosis.

## 2 MATERIALS AND METHODS

### 2.1 Constructing MCAO/R rat model

Sprague Dawley (SD) rats, 8‐9 weeks old, weighing between 260 g and 280 g, were obtained from Beijing Vital River Laboratory Animal Technology Co., Ltd. The rats were housed in animal laboratories with SPF‐grade conditions, including a humidity range of 60%–65% and a temperature range of 22‐25°C. The experiments commence after 1 week of adaptive feeding. The Institutional Animal Ethics Committee has approved the experimental procedure and plan for animal usage.

Transient cerebral ischemia‐reperfusion was induced in rats using the modified Zea Longa method for the focal cerebral ischemia‐reperfusion model (MCAO/R). Rats were anesthetized with an intraperitoneal injection of 2% sodium pentobarbital (30 mg/kg) and positioned supine on a rigid surface. After shaving and disinfecting the neck with 75% ethanol, a small incision was made at the neck's midline. The right, internal, and external carotid arteries were isolated with ophthalmic curved forceps, and the internal carotid artery was occluded using a 4‐0 nylon suture coated with poly‐L‐lysine. The suture was inserted to a depth of approximately 18–20 mL until the black mark on the suture aligned with the trigeminal nerve. Ischemia was induced for 1 h, followed by suture removal and a 24‐h reperfusion period. Sham‐operated animals underwent the same procedure without artery occlusion.^18^

### 2.2 High‐throughput transcriptome sequencing

Total RNA was extracted from rat brain tissues using Trizol reagent and assessed for quality with a Qubit® 2.0 Fluorometer and Agilent Bioanalyzer 2100, ensuring concentrations over 100 ng/μL and purity ratios of 1.8‐2.1. Libraries, created using the NEBNext® UltraTM RNA Library Prep Kit, were sequenced on the Illumina‐HiSeq 550 with 125/150 bp paired‐end reads. Quality control utilized FastQC and HISAT2 for genome alignment.^19^, ^20^

The quality of paired‐end reads in raw sequencing data should be assessed using FastQC software v0.11.8. The raw data were preprocessed using version 1.18 of the Cutadapt software to eliminate Illumina sequencing adaptors and poly(A) tail sequences. Eliminate reads with nitrogen (N) content exceeding 5% using a Perl script. Seventy percent of reads with a base quality above 20 were extracted using FASTX Toolkit version 0.0.13 software. Fix paired‐end sequences using BBMap software. Finally, the filtered high‐quality reads were aligned to the rat reference genome using version 0.7.12 of the HISAT2 software.^21^, ^22^

### 2.3 Data acquisition

The gene expression profiling dataset GSE163614, which consists of rat brain tissue samples after MCAO/R, was acquired from the Gene Expression Omnibus (GEO) database (https://www.ncbi.nlm.nih.gov/gds). The dataset included three samples of rat brain tissue from the Sham group and three from the MCAO/R group.^23^

### 2.4 Differential gene expression analysis

During the analysis of differential gene expression, we utilized high‐throughput transcriptome sequencing data (RNA‐seq) and the gene expression profile dataset GSE163614 from MCAO/R rat brain tissue. The differential gene expression was filtered using the “limma” package in the R language, applying a screening threshold of |logFC| > 1 and a *p* < 0.05. We produced a volcano plot and heatmap to visualize the differential gene expression. It was done using the “ggplot2” and “heatmaps” packages in the R programming language.^24^, ^25^

### 2.5 GO and KEGG functional enrichment analysis

The differentially expressed genes underwent GO and KEGG functional enrichment analysis using the R language packages, “clusterProfiler,” “org.Hs.eg.db,” “enrichplot,” and “ggplot2.” A bubble chart displaying the enrichment results was generated for three entries in the gene ontology (GO): biological process (BP), cellular component (CC), and molecular function (MF). Additionally, a circular plot was created to represent the enrichment analysis results of KEGG.^26^

### 2.6 Construct weighted gene co‐expression networks

The co‐expression network of RNA‐seq data and expression profile dataset GSE8609 was constructed using the WGCNA algorithm. A batch correction was performed using the “sva” package in the R language. We conducted WGCNA enrichment analysis using the ‘WGCNA’ package in R. The chip soft threshold was calculated using the R software, and gene modules and co‐expression matrix networks were constructed based on this threshold. The correlation between gene modules and clinical data was obtained using dynamic hybrid cutting, and the results were visualized as a heatmap. The modules correlated with CIRI were chosen for further analysis.^27^

### 2.7 Knock out RACK1 using the CRISPR/Cas9 gene editing system

The RACK1 gene was knocked out in SK‐N‐SH cells utilizing the CRISPR/Cas9 gene editing system (GeneChem, China, Shanghai). We designed sgRNA using Benchling's CRISPR Guide RNA Design Tools and selected sgRNA sequences with high scores and low off‐target rates. The forward primer sequence, Clone F, is 5′‐ACGGTGACTGTCTCTCCAGA‐3′ (PAM: AGG), and the reverse primer sequence, Clone R, is 5′‐GATAGCCTGTGTGGCCAATG‐3′ (PAM: TGG). These primer sequences should be cloned into the LentiCRISPR V2 lentiviral vector. Lentivirus packaging was accomplished by transfecting into 293T cells (CL‐0005, Wuhan Punois Life Science Co., Ltd.) at a multiplicity of infection (MOI) of 10, with a working titer of approximately 5 × 10^6^ PFU/mL. SK‐N‐SH cells were infected with a Lentiviral CRISPR/Cas9 vector and screened with 4 μg/mL puromycin (A1113803, Thermo) to create stable RACK1 knockout cells. It is illustrated in Figure S1. Viable cell cloning was conducted using the restricting dilution method. Primers (F: 5′‐TGAAGACCAACCACATTGGC‐3′; R: 5′‐CTTGCCTCCAGAAGCACAGA‐3′) were designed for validation of the RACK1 sgRNA recognition site through RT‐qPCR and Western blot experiments for future investigations.^28^

### 2.8 RACK1 complementation experiment

To complement RACK1 in RACK1 knockout cells, we designed three mutation sites and introduced synonymous mutations to prevent the targeting of sgRNA. The mutation sites are found in the sequence (GTATGGAACTTGGCTAACTGCAAGCTGAAGACGAACCACATTGGCCACACAGGCTATCTGAACACGGTGACTGTCTCTCCGGATGGATCCCTCTGTGCTTCTGGAGGTAAG) of exon 5 of RACK1. The GeneArt™ Site‐Directed Mutagenesis PLUS System (A14604, Invitrogen) was used to construct the plasmid, which was subsequently transfected into the cell line for complementation using Lipofectamine 3000 (L3000075, Invitrogen). The experiment is conducted according to the instructions provided in the reagent kit.^29^

### 2.9 Cell culture and oxygen‐glucose deprivation/reperfusion (OGD/R) treatment

Human neuroblastoma SK‐N‐SH cells (CL‐0214) from Wuhan PunoSai Life Technologies Co., Ltd. were cultured in Dulbecco's modified Eagle medium (DMEM) enriched with 10% fetal bovine serum and 1% penicillin‐streptomycin in a 5% CO_2_ incubator. For the oxygen‐glucose deprivation (OGD) experiment, cells were grown in glucose‐free DMEM until they reached about 70% confluence. They were then exposed to a 95% nitrogen and 5% CO_2_ atmosphere in a three‐gas incubator (Thermo Fisher Scientific) for 6 h. Post‐OGD, the cells were returned to glucose‐containing DMEM and maintained under normal culture conditions to fulfill experimental protocols.^30^

### 2.10 Cell treatments

SK‐N‐SH cell groups in the study included WT group (untreated wild‐type SK‐N‐SH cells), WT+OGD/R group (treated with OGD/R), RACK1‐KO group (CRISPR/Cas9 knockout of RACK1), RACK1‐KO+OGD/R group (RACK1 knockout with OGD/R treatment), RACK1‐KO+RACK1+OGD/R group (RACK1 knockout, complemented, then treated), RACK1‐KO+RACK1+OGD/R+Mdivi‐1 group (RACK1 complemented cells treated with 1 μM Mdivi‐1 and OGD/R), RACK1‐KO+RACK1+OGD/R+sh‐Parkin group (RACK1 knockout, complemented, infected with sh‐Parkin lentivirus, then OGD/R treated). Mdivi‐1 was purchased from Selleck (S7162, Shanghai, China).

Lentiviruses were packaged using pHAGE‐puro, pSPAX2, and pMD2.G plasmids in 293 T cells by Shenggong Biological Engineering, Shanghai, China. After 48 h, the supernatant was filtered and concentrated; two virus batches were mixed to assess the titer. SK‐N‐SH cells, seeded at 1 × 10^5^ per well, were infected with the lentivirus (MOI = 10, titer about 5 × 10^6^ TU/mL) and polybrene (5 μg/mL). After 4 h, the medium was diluted and replaced after 24 h. Puromycin was used for stable line selection. Table S1 details the viral sequences, and Figure S2 shows the silencing effects, guiding the selection of sequences for further experimentation.

### 2.11 RT‐qPCR

RNA was extracted from tissue and cells using Trizol, provided by Thermo Fisher Scientific, and analyzed for concentration and purity with the NanoDrop One/OneC, where the A260/A280 ratio was found to be 2.0 and concentration exceeded 5 μg/μL. cDNA synthesis was conducted using a kit from Beyotime (Shanghai). RT‐qPCR was performed with Vazyme Biotech's assay kit (Q511‐02) in a Bio‐rad CFX96 system, following a standard protocol of 40 cycles, starting with pre‐denaturation at 95°C for 30 s, denaturation at 95°C for 10 s, annealing at 60°C for 30 s, and extension at 72°C for 30 s. The melt curve analysis ranged from 65 to 95°C. Primer details are listed in Table S2.

The gene expression ratio between the experimental and control groups is 2^−ΔΔCt^, with GAPDH serving as the reference gene. The formula for ΔΔCt is as follows: ΔΔCt = ΔCt experimental group −ΔCt control group. Here, ΔCt represents the difference between the Ct values of the target gene and the reference gene. The experiment was repeated three times.^31^

### 2.12 Western blot

Total protein was extracted from tissues and cells using RIPA lysis buffer (Shanghai Beyotime, P0013B) supplemented with 1% PMSF, and mitochondrial and cytoplasmic proteins were isolated using the Beyotime mitochondrial isolation kit (C3601). Protein concentration was determined by the BCA assay (P0011, Beyotime Shanghai). SDS‐PAGE gels (8%‐12%) were prepared to separate proteins, which were then transferred to PVDF membranes (1,620,177, BIO‐RAD). Membranes were blocked with 5% skim milk and incubated overnight at 4°C with primary antibodies including anti‐RACK1 (ab129084, 1:1000), anti‐Bax (ab32503, 1:1000), anti‐Bcl2 (ab196495, 1:1000), anti‐caspase‐9 (ab2013, 1:1000), anti‐cleaved‐caspase‐3 (PA5‐114687, 1:500, Invitrogen), anti‐Cytochrome C (CytC) (ab133504, 1:2000), anti‐TOM20 (ab186735, 1:1000), anti‐COXIV (ab202554, 1:1000), anti‐VDAC1 (ab14734, 1:1000), anti‐PINK1 (ab186303, 1:1000), anti‐Parkin (ab77924, 1:1000), and anti‐beta Actin (ab8226, 1:1000) (all antibodies from Abcam). After washing with TBST, membranes were incubated with HRP‐conjugated secondary antibodies (ab6721 or ab6728, 1:2000) at room temperature. Enhanced chemiluminescence (Bio‐Rad ECL, 1705062) was used for detection on a GE Image Quant LAS 4000C gel imager. Protein expression levels were quantified by comparing grayscale values to beta‐actin, serving as an internal control.^29^ Each experiment was performed three times.

### 2.13 CCK‐8 assay

Cells were digested, resuspended to a concentration of 1 × 10^5^ cells per milliliter, and seeded at 100 μL per well in a 96‐well plate. They were incubated overnight under standard conditions, treated with a CCK‐8 kit (C0037; Beyotime, Shanghai, China), and subjected to a 6‐h OGD treatment. Cell viability was assessed at 3, 6, 12, and 24 h post‐reperfusion using the CCK‐8 method. For each measurement, 10 μL of CCK‐8 detection solution was added and incubated for 4 h in a CO_2_ incubator. The absorbance at 450 nm was measured using a microplate reader, and growth curves were plotted.^32^

### 2.14 Flow cytometry

Cell apoptosis was detected using the Annexin V‐FITC/Sodium Iodide (PI) Apoptosis Detection Kit (C1062M, Beyotime, Shanghai, China). Cells were washed with cold PBS, resuspended in 195 μL of Annexin V‐FITC binding buffer, and supplemented with 5 μL of Annexin V‐FITC and 10 μL of propidium iodide. Following a 15‐min incubation at room temperature in the dark, apoptosis was quantitatively measured using flow cytometry with the FACSVerse system (BD, USA).^33^

### 2.15 ROS detection

ROS levels were measured using the ROS assay kit (S0033M, Beyotime, Shanghai, China). Cells were incubated in a 10‐μM DCFH‐DA solution at 37°C for 20 min, with periodic inversion every 3‐5 min to ensure uniform exposure. After incubation, cells were rinsed thrice with a serum‐free medium to remove excess DCFH‐DA. ROS detection was then performed using flow cytometry (FACSVerse, BD, USA).^34^

### 2.16 ATP content

ATP content was assessed using the ATP content detection kit (S0026, Beyotime, Shanghai, China). The culture medium was removed, and cells in a six‐well plate were lysed with 200 μL of lysis buffer; tissues were treated with 800 μL post‐MCAO/R. The lysates were pipetted repeatedly for thorough mixing and then centrifuged at 4°C and 12,000 × g for 5 min to obtain the supernatant. ATP concentration was measured by constructing a standard curve per the kit's instructions. Additionally, protein concentrations were determined using the BCA protein assay kit to calculate and document the ATP content for each group accurately.^35^

### 2.17 LDH release

In accordance with the instructions provided by the LDH test kit (C0017, Beyotime, Shanghai, China), the cells and tissues to be tested should be collected and thoroughly lysed. Subsequently, the assay solution should be added to the sample. The samples were incubated at room temperature for 30 min, followed by measurement of absorbance at 490 nm using a multimode microplate reader to determine LDH release.^36^

### 2.18 JC‐1 detection of mitochondrial membrane potential

Mitochondrial membrane potential was measured using the JC‐1 kit (C2003S, Beyotime, Shanghai, China). Cells were trypsinized, resuspended in 0.5 mL medium, and stained with JC‐1. After mixing, the cells were incubated at 37°C for 20 min and then centrifuged at 600 × g for 4 min to collect the pellet. The precipitate was washed with JC‐1 buffer, and mitochondrial membrane potential was assessed using flow cytometry (FACSVerse, BD, USA). Additionally, mitochondria were extracted from rat brains post‐24 h of MCAO/R, stained with JC‐1, and analyzed with a multimode enzyme label reader (SpectraMax® iD5, Molecular Devices, USA).^37^

### 2.19 Observe co‐localization of mitochondria and autophagosomes

The pHBmTur‐Mito adenovirus from Hanheng Biotechnology (Shanghai, China) and the pCMV‐mCherry‐GFP‐LC3B plasmid (D2816‐1 μg, Beyotime, Shanghai, China) were used to target mitochondria and autophagosomes, respectively. SK‐N‐SH cells were transfected with the plasmid using Lipo3000 liposomes (L3000075, Invitrogen), followed by adenovirus introduction at a multiplicity of infection (MOI) of 20. Cells underwent OGD/R treatment 48 h post‐transfection and were subsequently fixed with 4% formaldehyde and stained with DAPI. Localization of mitochondria and autophagosomes was visualized using a laser scanning confocal microscope (LSM 800, Zeiss, Germany).^34^

### 2.20 TEM observation of autophagosomes

In vivo experiments isolated rat brains and sliced the ischemic cerebral cortex into 1 mm^3^ pieces, subsequently fixed in 2% paraformaldehyde. In vitro experiments involve harvesting cells after the OGD/R treatment. Following PBS washes, the cells were fixed in a 2% glutaraldehyde solution. The samples were sliced and double stained with slice and uranium lead, then observed and photographed using a Japan Hitachi TEM system (HT7800).^38^

### 2.21 Immunofluorescent staining

Cells were digested, counted, and cultured in immunofluorescence chambers at a density of 2 × 10^5^ cells per well. After reaching approximately 90% confluence, the cells were washed with ice‐cold PBS and fixed with 4% paraformaldehyde for 15 min at room temperature. Following a triple PBS wash, cells were blocked with 5% BSA for 30 min. They were then incubated overnight at 4°C with anti‐COXIV (1:200, ab202554, UK) and anti‐TOM20 (1:200, ab186735, UK) in BSA. After further washing, the cells were incubated with FITC‐conjugated anti‐rabbit IgG (ab6717, Abcam, UK) and Alexa Fluor® 647‐conjugated anti‐rabbit IgG (ab150083, Abcam, UK) at room temperature in the dark for 1 h. Post‐incubation, cells were washed and stained with DAPI, followed by a final wash and application of a fluorescence quencher. Slides were sealed and imaged using an LSM 800 confocal microscope (Zeiss, Germany), and the images were quantitatively analyzed with Image‐Pro Plus 6.0 software.

### 2.22 Animal experiment

RACK1 siRNA (5′‐GCGTCTCGAGACAAGACCATCATCA‐3′) and oe‐RACK1 were chemically synthesized by GenePharma (Shanghai, China) and prepared at a concentration of 2 μg/μL in RNase‐free water. The rats were anesthetized by intraperitoneal injection of 2% pentobarbital sodium (30 mg/kg). They were then positioned in a stereotaxic apparatus (69100, RWD Life Science, China, Shenzhen) to expose the bregma point, which corresponds to the intersection of the sagittal and coronal sutures on the skull. A 25‐μL Hamilton syringe was used to inject 15 μL of either RACK1 siRNA or oe‐RACK1 solution into the left lateral ventricle at coordinates 1.0 mm posterior, 2.0 mm lateral, and 3.5 mm deep relative to the bregma at a rate of 1 μL/min. The needle was maintained in place for 10 min before withdrawal. MCAO/R surgery followed 24 h post‐injection, with Mdivi‐1 (10 mg/kg) administered at reperfusion onset.

Animal groups included: 1. Sham group (undergoing sham surgery), 2. MCAO/R group (undergoing MCAO/R surgery to construct CIRI model), 3. MCAO/R+si‐NC+oe‐NC group (injected with si‐NC+oe‐NC plasmids into the lateral ventricle before MCAO/R surgery), 4. MCAO/R + si‐RACK1 group (injected with si‐RACK1 plasmids into the lateral ventricle before MCAO/R surgery), 5. MCAO/R + oe‐RACK1 group (injected with oe‐RACK1 plasmids into the lateral ventricle before MCAO/R surgery), 6. MCAO/R+oe‐RACK1+Mdivi‐1 group (injected with oe‐RACK1 plasmids and Mdivi‐1 into the lateral ventricle before MCAO/R surgery), with six rats per group. The study protocol was approved by the hospital's Animal Ethics Committee, and the experimental timeline is documented in Figure S3.

### 2.23 TTC staining

To determine the infarct volume, rats were anesthetized with sodium pentobarbital. Rat brain tissue samples were collected within 24 h after reperfusion and frozen at −20°C for 20 min. Next, the brain is sliced into 2‐mL‐thick sections and then incubated in a 2% TTC solution (CAS NO: 298‐96‐4, Merck, Germany) at a temperature of 37°C in a dark environment for 15‐30 min. Following this, we fixed it using a solution of 4% paraformaldehyde at 4°C for 48 h. A red signal indicates the presence of normal tissue, while a white signal indicates the presence of necrotic tissue. We capture sliced images with a digital camera and analyze them using ImageJ analysis software. The formula used to calculate the percentage of corrected infarct volume is as follows: {[Total infarct lesion volume − (Volume of the same hemisphere − Volume of the opposite hemisphere)]/Volume of the opposite hemisphere} × 100.^39^

### 2.24 Neurological impairment score

The Modified Neurological Severity Score (mNSS) is utilized to assess the extent of neurological damage in rats. The assessment evaluates the motor function, with a maximum score of 6 points. It includes observing the animal's touch avoidance, gait, and climbing behavior. Inadequate performance in these activities leads to an elevated score. Sensory and visual reactions, which are assessed on a scale of 2, are evaluated by testing the animals' tactile and visual responses. Impaired responses will result in an increased score during the assessment of balance testing, which has a maximum score of 6 points. This assessment involves observing if the animal can maintain balance and normal posture while suspended. Impaired balance ability will result in higher scores during reflex evaluation (with a maximum score of 4), including cortical reflexes and tentacle reflexes. If the reflection is damaged, the score will increase. The total potential score is 18 points, and it is assessed by two researchers who are unaware of this study. A higher score signifies a more severe injury. Twenty‐four hours after ischemia treatment, we obtained mNSS scores for each rat.^34^

### 2.25 TUNEL staining

Following fixation, dehydration, and embedding in paraffin, the rat brain tissues from each group were sectioned into 3‐mm‐thick slices and then subjected to baking at 72°C for 1 h. High‐pressure repair of antigens was carried out during the dewaxing and hydration process. After incubation with 3% hydrogen peroxide, the sections were incubated overnight at 4°C with a NeuN antibody (ab177487, diluted at 1:100, Abcam, USA). It was followed by incubation at room temperature for 1 h with a goat anti‐mouse Alexa Fluor 594 secondary antibody (ab150080, diluted at 1:500, Abcam, USA). Each tissue slice was incubated in 50 μL of TUNEL detection solution (C1086, Beyotime, Shanghai, China) at 37°C in darkness for 1 h. Following nuclear staining, observations were made using a laser scanning confocal microscope (LSM 800, Zeiss, Germany).^40^

### 2.26 Statistical analysis

This study utilized R software version 4.2.1, compiled using the RStudio integrated development environment. The specific version of RStudio employed was 4.2.1. The file processing should be performed using Perl, specifically version 5.30.0. The cytoscape version is 3.7.2, and all bar charts were created using GraphPad Prism 8.0. Quantitative data were expressed as mean ± standard deviation. All data were tested for normal distribution using the Shapiro‐Wilk test. Data conforming to normal distribution were analyzed using the independent samples *t*‐test to compare differences between the two groups, while data not normally distributed were analyzed using the Mann‐Whitney *U* test. Differences between groups were assessed using one‐way ANOVA, and differences among groups at different time points were analyzed using repeated measures ANOVA. Post hoc testing was performed using the Bonferroni method.^41^, ^42^

## 3 RESULTS

### 3.1 High‐throughput transcriptome sequencing identified the molecular characterization in MCAO/R rats

**FIGURE 1:**
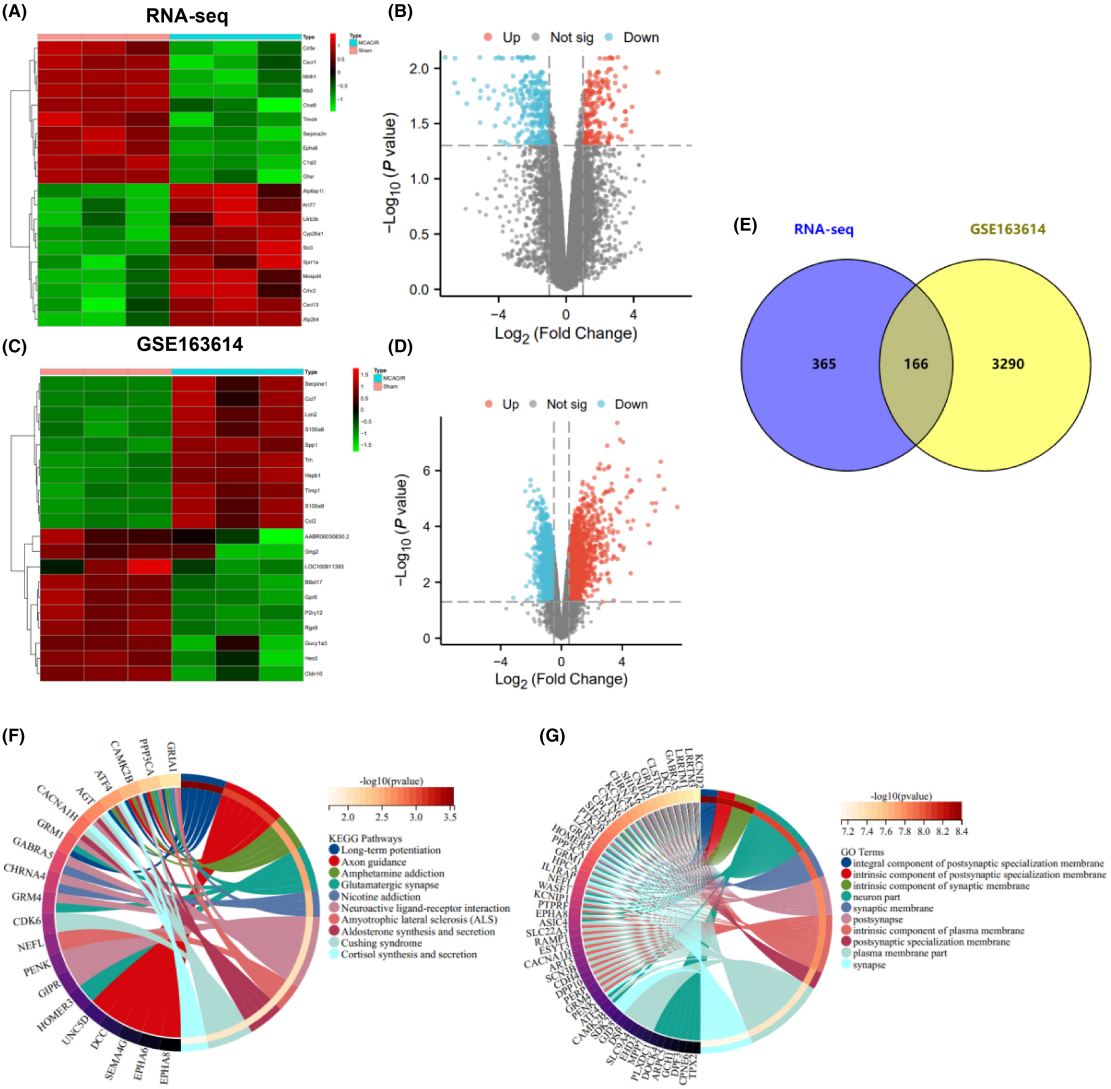
Differential expression and molecular characterization of genes in MCAO/R rats identified by high‐throughput transcriptome sequencing. (A) Heatmap displaying the top 10 highly expressed genes and the top 10 lowly expressed genes based on logFC in the MCAO/R rat brain tissue from high‐throughput transcriptome sequencing; (B) Volcano plot displaying the upregulated and downregulated differentially expressed genes in the MCAO/R rat brain tissue from high‐throughput transcriptome sequencing (sham *n* = 3, MCAO/R *n* = 3); (C) Heatmap displaying the top 10 highly expressed genes and the top 10 lowly expressed genes based on logFC in the MCAO/R rat brain tissue from the GSE163614 dataset; (D) Volcano plot displaying the upregulated and downregulated differentially expressed genes in the MCAO/R rat brain tissue from the GSE163614 dataset (sham *n* = 3, MCAO/R *n* = 3); (E) Venn diagram showing the intersection of differentially expressed genes between the RNA‐seq data and the GSE163614 dataset; (F) KEGG enrichment analysis of the 166 intersecting differentially expressed genes; (G) GO enrichment analysis of the 166 intersecting differentially expressed genes.

To investigate the molecular mechanisms involved in brain ischemia‐reperfusion injury and provide guidance for clinical medication, we established the CIRI rat model through MCAO/R surgery. We then conducted high‐throughput transcriptome sequencing to analyze the transcriptome of brain tissues in both the MCAO/R and Sham groups. The heat map illustrates the top 10 differentially expressed genes based on the logFC (fold change) (Figure 1A), whereas the volcano plot indicates a total of 206 upregulated genes and 325 downregulated genes (Figure 1B). Furthermore, the gene expression profile of brain ischemia‐reperfusion injury in the MCAO/R rat model was discovered in the GEO database. This profile is depicted as a heat map and volcano plot, illustrating the differentially expressed genes (Figure 1C,D). Furthermore, 166 differentially expressed genes were identified when combined with RNA‐seq data (Figure 1E).

Using KEGG and GO enrichment analysis, we identified that the differentially expressed genes were predominantly enriched in neuro‐related signaling pathways such as long‐term potentiation, axon guidance, amphetamine addiction, and glutamatergic synapse (Figure 1F). Furthermore, the gene ontology (GO) analysis indicated that these 166 differentially expressed genes potentially involve synaptic and neuronal‐related components (Figure 1G).

The comprehensive analysis above identified 166 differentially expressed genes and further revealed their involvement in neuro‐related signaling pathways.

### 3.2 WGNCA analysis identified core genes associated with CIRI

**FIGURE 2:**
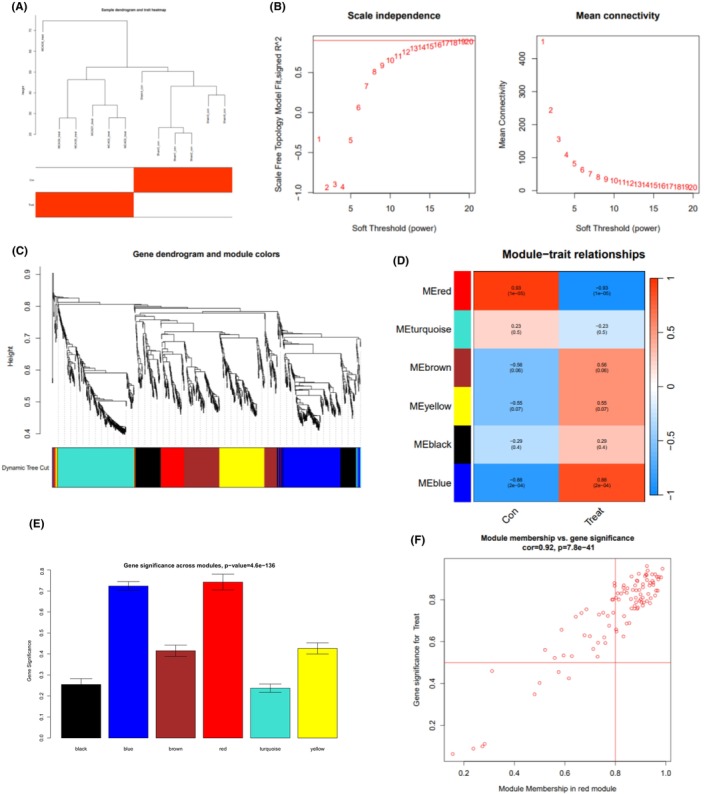
Co‐expression gene module identification related to CIRI by WGCNA analysis. (A) Sample clustering analysis based on RNA‐seq and GSE163614 datasets; (B) Analysis of the scale‐free fitting index (left) and average connectivity (right) for various soft‐thresholding powers β; (C) Hierarchical clustering dendrogram of co‐expression genes, where each leaf on the dendrogram corresponds to a gene module and each color represents a gene module; (D) Heatmap of the Pearson correlation coefficients between the modules and the HCC phenotype, with each cell containing the corresponding correlation coefficient and *p*‐value; (E) Significance analysis of gene sets between modules; (F) Scatter plot of gene significance versus module membership (MM) for the genes in the red module.

Initially, batch correction was done on the merged RNA‐seq data and the microarray dataset GSE163614. Subsequently, sample‐level clustering was performed using the “WGCNA” package in R software (Figure 2A). Subsequently, topological calculations were undertaken, and it was determined that a soft threshold of *β* = 16 best facilitated the construction of a scale‐free network (Figure 2B). The correlation matrix is transformed into an adjacency matrix using a soft threshold. Subsequently, it is converted into a topological overlap matrix (TOM). We employed hierarchical clustering analysis with average linkage to classify the relevant modules and guarantee a minimum of 50 genes in each module (Figure 2C). In total, six co‐expression modules were identified (Figure 2D). Additional calculations were performed to ascertain the correlation between genes and CIRI, demonstrating that the red module displayed the most robust correlation with CIRI.

Consequently, the red module was identified as the core module (Figure 2E). Gene significance (GS) and module membership (MM) calculations were conducted on 132 genes within the red module. A scatter plot illustrating the correlation was generated, demonstrating a distinct positive relationship between GS and MM (Figure 2F).

GO and Kyoto Encyclopedia of Genes and Genomes (KEGG) enrichment analyses were conducted on the hub genes within the 132 red modules. The results of the KEGG analysis indicated that these genes were primarily enriched in various signaling pathways, including the neuroactive ligand‐receptor interaction pathway, the calcium signaling pathway, and the cAMP signaling pathway (Figure S4A). The results of the GO analysis demonstrated that these genes are primarily enriched in regulating multicellular organismal processes, response to external stimuli, and nervous system development, along with other biological processes (Figure S4B). Moreover, they are also enriched in cellular components associated with nervous system development, integral components of the membrane, and plasma membrane parts (Figure S4C). Additionally, they exhibit molecular functions like signaling receptor binding and signaling receptor activity (Figure S4D).

In conclusion, our WGNCA analysis successfully identified co‐expression gene modules correlated with CIRI. Additionally, we discovered 132 genes that are closely related to CIRI. Further enrichment analysis unveiled the essential roles of these genes in neuro‐related signaling pathways and biological processes, offering substantial clues for studying the molecular mechanisms behind brain ischemia‐reperfusion injury.

### 3.3 Deciphering the role of RACK1 in cerebral ischemia‐reperfusion injury: from gene intersection analysis to functional validation in vitro

**FIGURE 3:**
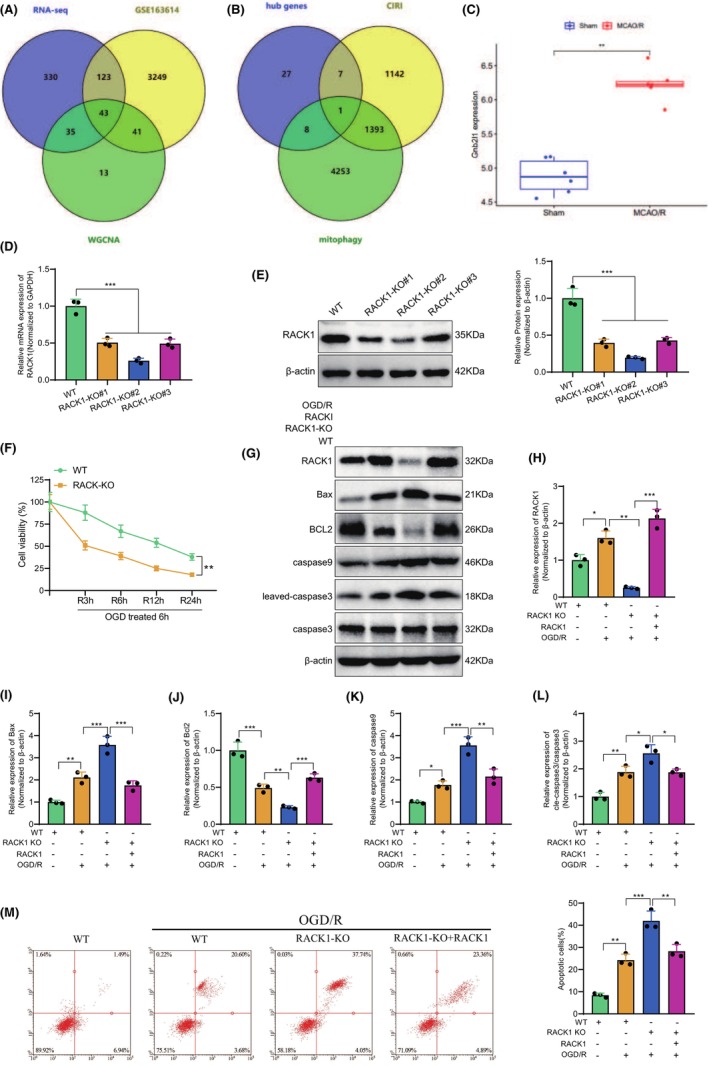
The effect of RACK1 on OGD/R‐induced cell apoptosis. (A) Venn diagram showing the intersection of differentially expressed genes from RNA‐seq, GSE163614, and WGCNA analysis for co‐expression module genes; (B) Venn diagram showing the intersection of CIRI‐related genes and mitophagy‐related genes, with further intersection of 43 common genes to obtain the unique intersecting gene RACK1; (C) Expression profiles of RACK1 in the Sham group and the MCAO/R group in the RNA‐seq data and GSE163614 dataset (Sham *n* = 6, MCAO/R *n* = 6); (D) qRT‐PCR detection of RACK1 mRNA levels in different groups of cells; (E) Western blot analysis of RACK1 protein expression levels in different groups of cells; (F) CCK8 assay to assess cell viability changes in different groups after OGD/R treatment at different time points; (G) Western blot analysis of RACK1, Bax, Bcl2, caspase‐9, and cleaved‐caspase‐3 protein expression levels in different groups of cells; (H–L) Semi‐quantitative analysis of RACK1, Bax, Bcl2, caspase‐9, and cleaved‐caspase‐3 protein expression levels; (M) Flow cytometry analysis of cell apoptosis in different groups. *p* < 0.05, *p* < 0.01, **p < 0.001. The cell experiments were repeated three times.

To identify the key genes associated with CIRI, we conducted an additional intersection between the core genes obtained from the WGCNA analysis and the differentially expressed genes, which resulted in 43 overlapping genes (Figure 3A). To investigate the connection between CIRI and mitophagy, we retrieved relevant genes from the GeneCards database. By comparing these genes with a set of 43 intersected genes (Figure 3B), we identified a unique gene, RACK1 (Gnb2l1). We also observed increased RACK1 expression in rat brain tissue following MCAO/R surgery (Figure 3C). This finding further underscores the crucial role of RACK1 in CIRI.

To investigate the function of RACK1 in CIRI, we performed in vitro experiments using SK‐N‐SH cells. Following OGD6h treatment, we measured RACK1 expression at several reperfusion time points, specifically 3, 6, 12, and 24 h. We found that RACK1 expression increased after 3 h of reperfusion but gradually decreased with longer reperfusion times (Figure S5A,B). We employed CRISPR/Cas9 technology to effectively disrupt the RACK1 gene, enabling the generation of stable RACK1‐knockout cell lines. Multiple RACK1‐KO cell clones were selected, and the expression of RACK1 was analyzed using qRT‐PCR and Western blot. The results demonstrated that the knockout of RACK1 in RACK1‐KO#2 cells had the most pronounced effect (Figure 3D,E). Therefore, we chose RACK1‐KO#2 cells (RACK1‐KO) for further experiments. To investigate the impact of RACK1 on cell viability, we exposed the cells to 6 h of OGD treatment followed by reperfusion. We assessed cell viability using the CCK‐8 assay. The results indicate a decrease in cell viability after OGD/R treatment, with a further reduction observed in RACK1 KO cells (Figure 3F). To construct an in vitro model of cerebral ischemia‐reperfusion injury (CIRI), we selected OGD treatment for 6 h followed by 3 h of reperfusion due to the rapid decrease in cell viability during this period.

Moreover, the expression levels of apoptotic proteins related to the mitochondrial pathway were assessed using Western blot analysis in the cells. The results indicated that following OGD/R treatment, there was an increase in the expression of pro‐apoptotic proteins Bax, caspase‐9, and cleaved‐caspase‐3. Conversely, the expression of the anti‐apoptotic protein BCL2 was suppressed. Furthermore, these alterations are even more prominent in RACK1 knockout (KO) cells. However, they are reverted upon RACK1 complementation (Figure 3G–L).

Furthermore, flow cytometry analysis confirmed these findings. OGD/R treatment markedly induced cell apoptosis, with a higher apoptosis rate observed in RACK1 KO cells than in WT cells. However, upon reintroduction of RACK1, the apoptosis rate in RACK1 KO cells decreased (Figure 3M).

In conclusion, our findings indicate that knockout of RACK1 may enhance OGD/R‐induced apoptosis through the mitochondrial pathway, while reintroducing RACK1 could undo this effect. These findings offer crucial insights into the ongoing exploration of RACK1's role in CIRI.

### 3.4 Elucidating the protective role of RACK1 in mitochondrial function and apoptosis during cerebral ischemia‐reperfusion injury

**FIGURE 4:**
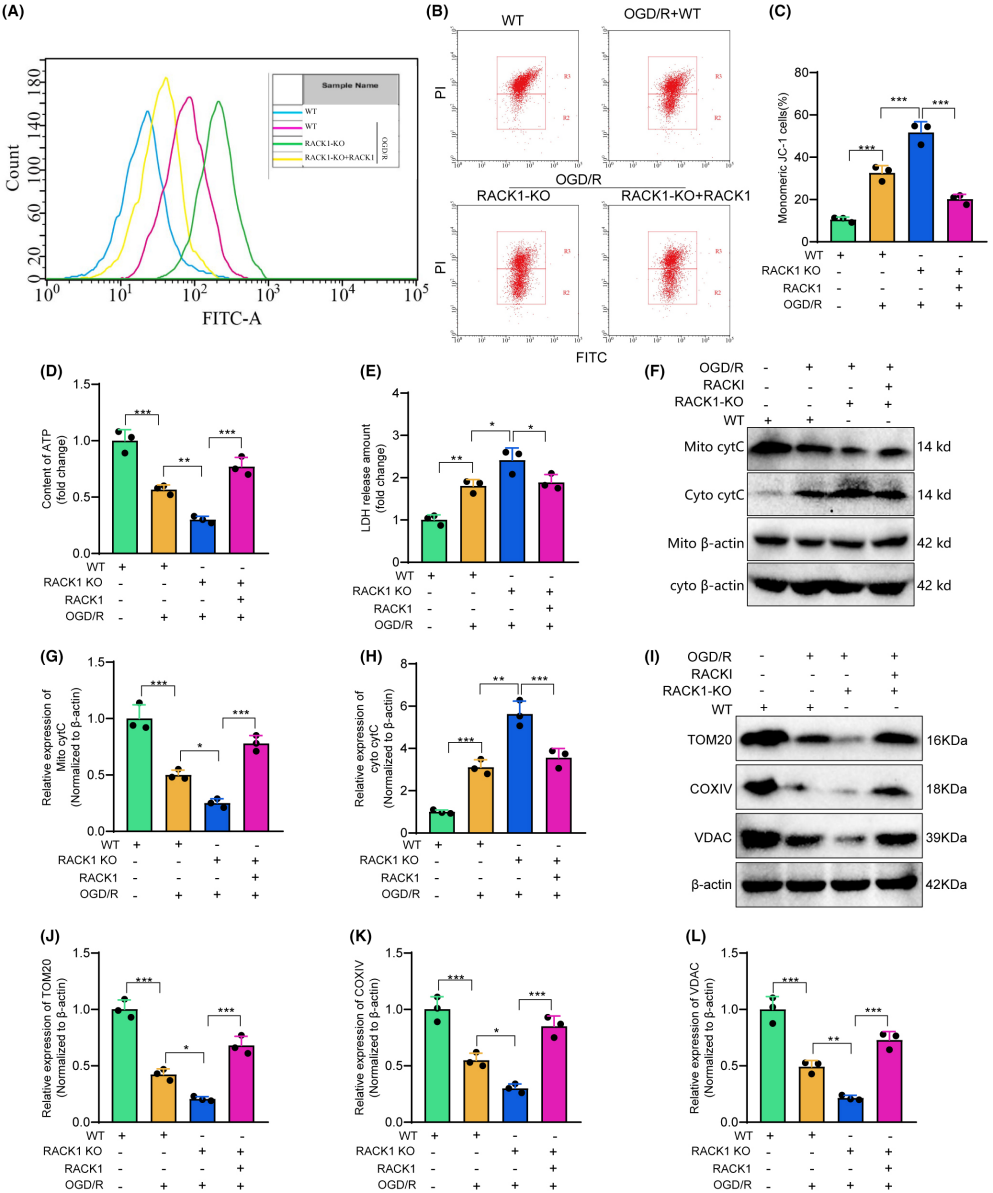
The effect of RACK1 on OGD/R‐induced cell apoptosis through mitochondrial function. (A) Measurement of ROS levels in cells of different groups using ROS detection reagent; (B) Measurement of mitochondrial membrane potential in cells of different groups using JC‐1 staining; (C) Quantification of cells with monomeric JC‐1 staining; (D, E) Detection of ATP levels (D) and LDH release (E) in cells of different groups using respective assay kits; (F) Western blot analysis of cytochrome C expression changes in mitochondria and cytosol of cells from different groups; (G) Statistical analysis of mitochondrial cytochrome C changes; (H) Statistical analysis of cytosolic cytochrome C changes; (I) Western blot analysis of TOM20, COXIV, and VDAC expression changes in cells of different groups; (J–L) Semi‐quantitative analysis of TOM20, COXIV, and VDAC expression levels. *p* < 0.05, *p < 0.01. The cell experiments were repeated three times.

Prior studies have demonstrated that the knockout of RACK1 enhances cell apoptosis triggered by oxygen‐glucose deprivation and reoxygenation (OGD/R). To further investigate the mechanism of RACK1, we directed our attention toward determining whether RACK1 exerts its anti‐apoptotic effects through enhancing mitochondrial function. Given that aged or damaged mitochondria may contribute to generating toxic ROS during IR injury, we initially assessed the levels of ROS in cells from each group using a ROS detection kit. The results indicated that OGD/R treatment elevated the levels of reactive oxygen species (ROS), and the knockout of RACK1 further exacerbated the accumulation of ROS. On the other hand, the reintroduction of RACK1 led to a substantial decrease in the levels of ROS (Figure 4A). To further assess mitochondrial function, we employed JC‐1 staining to measure the membrane potential of mitochondria in cells. The results demonstrate that following OGD/R treatment, there is a decrease in mitochondrial membrane potential and an increase in the number of cells labeled with JC‐1 monomers, which indicates an elevated number of damaged mitochondria. The knockout of RACK1 exacerbates this phenomenon even further, but the restoration of RACK1 rescues the increase in mitochondrial membrane potential and decreases the number of damaged mitochondria (Figure 4B,C). Additional analysis of ATP content and LDH release in cells demonstrated that following OGD/R treatment, there was a decrease in ATP content and an increase in LDH release. Knocking out RACK1 further intensified this effect, whereas replenishing RACK1 resulted in an increase in ATP content and a decrease in LDH release (Figure 4D,E).

Damaged mitochondria release cytochrome C into the cytoplasm, thus facilitating cellular apoptosis.^34^ Additionally, we quantified the levels of cytochrome C in both the mitochondria and cytoplasm. The results demonstrated an increase in the expression of cytochrome C in the cytoplasm following OGD/R treatment. Additionally, OGD/R treatment enhanced cytochrome C's release from the mitochondria. Disrupting RACK1 further enhances the mitochondria‐mediated release of cytochrome C, resulting in an amplified cytoplasmic accumulation of cytochrome C. In contrast, the introduction of RACK1 suppressed the release of cytochrome C from the mitochondria (Figure 4F–H).

Finally, the levels of three crucial mitochondrial functional proteins, TOM20, COXIV, and VDAC, were examined using Western blot analysis to observe mitochondrial homeostasis. Our study found that treatment with OGD/R decreased TOM20, COXIV, and VDAC expression levels. Moreover, the knockout of RACK1 further reduced the expression levels of these three proteins. Nevertheless, replenishing RACK1 resulted in the observed change reversal (Figure 4I‐L). Furthermore, the conclusion was further supported by the immunofluorescence results of TOM20 and COXIV (Figure S6A,B).

In summary, the results from our experiment demonstrate the crucial role of RACK1 in preserving mitochondrial function during I/R injury. RACK1 may enhance mitochondrial function by reducing the accumulation of ROS, enhancing mitochondrial membrane potential, and inhibiting the release of cytochrome C. As a result, it reduces apoptosis in mitochondria‐mediated cell death.

### 3.5 RACK1 promotes mitophagy to enhance mitochondrial function and suppress apoptosis in cerebral ischemia‐reperfusion injury: modulatory effects of Mdivi‐1

**FIGURE 5:**
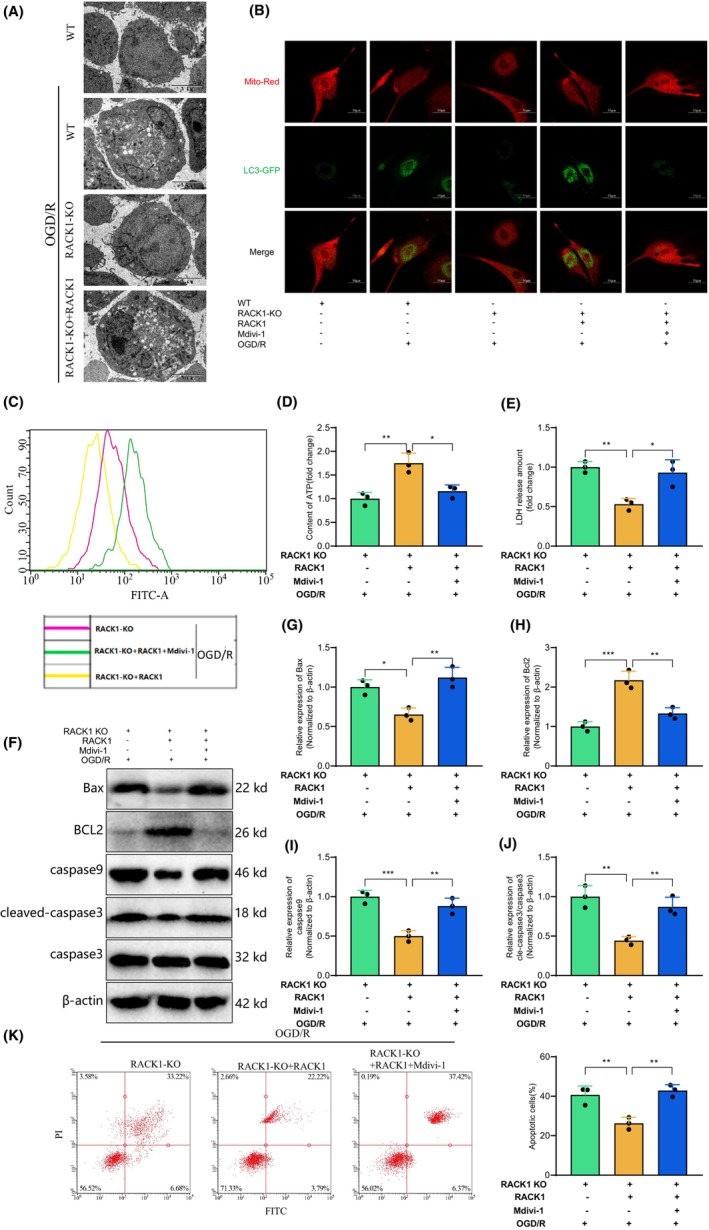
The effect of RACK1 on mitochondrial function and cell apoptosis through mitophagy. (A) TEM observation of mitochondrial autophagosomes in cells of different groups (bar = 1 μm); (B) Immunofluorescence co‐localization of mitochondria and autophagosomes; (C) Measurement of ROS levels in cells of different groups using ROS detection reagent; (D) Measurement of ATP levels in cells of different groups; (E) Measurement of LDH release in cells of different groups; (F) Western blot analysis of Bax, Bcl2, caspase‐9, and cleaved‐caspase‐3 protein expression levels in cells of different groups; (G–J) Semi‐quantitative analysis of Bax, Bcl2, caspase‐9, and cleaved‐caspase‐3 protein expression levels; (K) Flow cytometry analysis of cell apoptosis in different groups. *p* < 0.05, *p < 0.01. The cell experiments were repeated three times.

To investigate the impact of RACK1 on mitochondrial function and cell apoptosis through mitophagy, we first observed changes in cellular mitochondrial structure and mitophagy using transmission electron microscopy (TEM). The results indicate that following OGD/R treatment, mitochondria experienced swelling and subsequent cell death, while some mitochondria underwent autophagy. Nevertheless, we observed an increase in mitochondrial fission in RACK1 KO cells, while there was minimal occurrence of mitochondrial autophagy. Nevertheless, replenishing RACK1 resulted in a decrease in mitochondrial damage and an increase in mitochondrial autophagy (Figure 5A). In addition, we use Mdivi‐1 to inhibit mitophagy.^38^ And co‐localization of autophagosomes and mitochondria was observed. The results demonstrate increased mitochondrial autophagy in WT cells following OGD/R treatment. However, the knockout of RACK1 inhibits the OGD/R‐induced increase in mitochondrial autophagy. However, replenishing RACK1 enhanced mitochondrial autophagy, whereas subsequent treatment with Mdivi‐1 inhibited mitochondrial autophagy (Figure 5B).

Subsequently, we quantified the levels of ROS and adenosine triphosphate (ATP) and examined the release of lactate dehydrogenase (LDH) in different groups of RACK1 knockout (KO) cells. This analysis aimed to investigate whether the effects of RACK1 on enhancing mitochondrial function and suppressing cell apoptosis are modulated by mitophagy. The results demonstrated that in RACK1 knockout cells, the addition of RACK1 reduced the accumulation of ROS and the release of LDH while increasing the release of ATP. Nevertheless, this effect could be reversed through additional treatment with Mdivi‐1 (Figure 5C‐E). Western blot analysis revealed that reintroducing RACK1 in RACK1 knockout (KO) cells downregulated the expression of pro‐apoptotic proteins Bax, caspase‐9, and cleaved caspase‐3, while upregulating the expression of anti‐apoptotic protein Bcl‐2. However, treatment with Mdivi‐1 could also reverse this effect (Figure 5F–J). Furthermore, the detection of cell apoptosis using flow cytometry further supports our conclusion (Figure 5K).

Thus, these experimental results further prove RACK1's role in enhancing mitochondrial function and suppressing cell apoptosis by promoting mitophagy.

### 3.6 In vivo validation of RACK1's protective role in mitochondrial function, mitophagy, and neuronal survival in cerebral ischemia‐reperfusion injury

We have confirmed through a series of in vitro experiments that RACK1 plays a crucial role in modulating mitophagy, regulating mitochondrial function, and inducing cell apoptosis. We designed and conducted a series of in vivo experiments to further validate our research findings.

**FIGURE 6:**
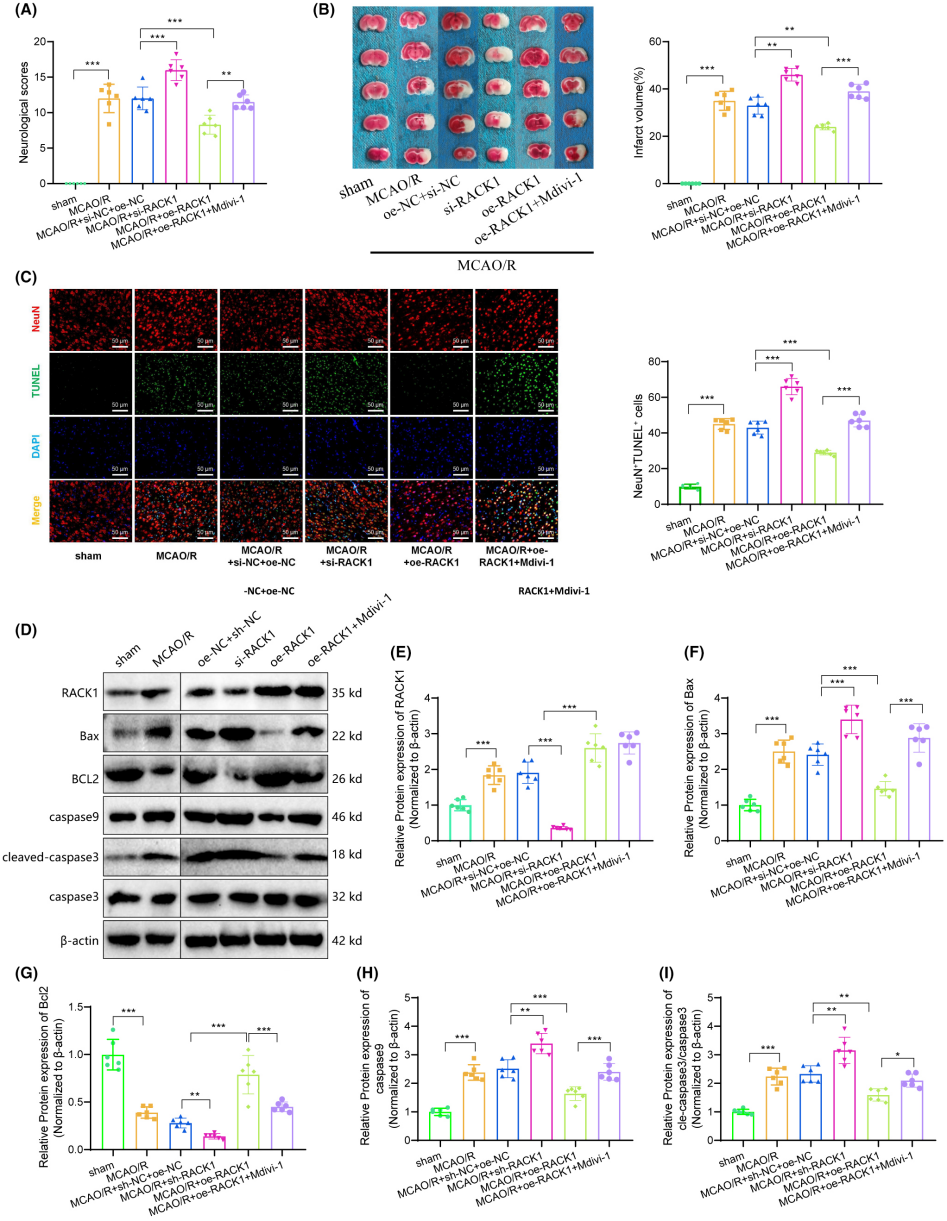
The effect of RACK1 on cell apoptosis and brain injury induced by MCAO/R. (A) Neurological damage scores of rats in different groups; (B) Measurement of ischemic brain area in rats of different groups using TTC staining; (C) Co‐staining of NeuN and TUNEL for the assessment of neuronal apoptosis; (D) Western blot analysis of RACK1, Bax, Bcl2, caspase‐9, and cleaved‐caspase‐3 protein expression levels in cells of different groups; (E–I) Semi‐quantitative analysis of RACK1, Bax, Bcl2, caspase‐9, and cleaved‐caspase‐3 protein expression levels. *p* < 0.05, *p < 0.01. Each group consisted of six rats.

Before conducting middle cerebral artery occlusion/reperfusion (MCAO/R) surgery in rats, we used techniques to silence or overexpress RACK1. We achieved overexpression or silencing of these genes by injecting plasmids into the lateral ventricles of the rats. Initially, we assessed the neurological function deficit score and infarct volume in each group of rats. The results suggest that rats' infarct volume and neurologic deficit score increased following MCAO/R surgery. Moreover, injection of si‐RACK1 resulted in a further increase in both the infarct volume and neurologic deficit score in rats. In contrast, overexpression of RACK1 led to a reduction in both the infarct volume and neurologic deficit score in rats. Notably, the counteraction of this decline is observed when Mdivi‐1, a mitochondrial fission inhibitor, is injected, suggesting that RACK1 exerts its protective effect against MCAO/R‐induced brain injury by modulating mitochondrial autophagy (Figure 6A,B).

Subsequently, neurons were labeled using anti‐NeuN antibodies, and neuronal apoptosis in the rat brain tissues of each group was observed through TUNEL staining. Compared to the sham group, the MCAO/R group of rats increased the number of apoptotic neurons in brain tissue. The RACK1‐silenced group of rats demonstrated an increase in the number of apoptotic neurons compared to their respective control groups, whereas rats overexpressing RACK1 exhibited a reduction in apoptotic neuron count. However, this reduction was offset after the injection of Mdivi‐1. These findings support our hypothesis that RACK1 plays a protective role in MCAO/R‐induced brain injury through its impact on mitochondrial autophagy (Figure 6C).

Furthermore, utilizing Western blot analysis, the expression of mitochondrial apoptosis‐related proteins Bax, Bcl‐2, caspase‐9, and cleaved‐caspase‐3 in the brain tissues of each group of rats was examined. It was observed that compared to the control group, the MCAO/R group showed increased expression of Bax, caspase‐9, and cleaved‐caspase‐3, while Bcl‐2 expression decreased. In rats with silenced RACK1, increased expression of Bax, caspase‐9, and cleaved‐caspase‐3, accompanied by decreased Bcl‐2 expression, was observed compared to the control group. However, in rats overexpressing RACK1, decreased expression of Bax, caspase‐9, and cleaved‐caspase‐3, along with increased Bcl‐2 expression, was noted, and these changes were reversed post‐Mdivi‐1 injection (Figure 6D–I).

**FIGURE 7:**
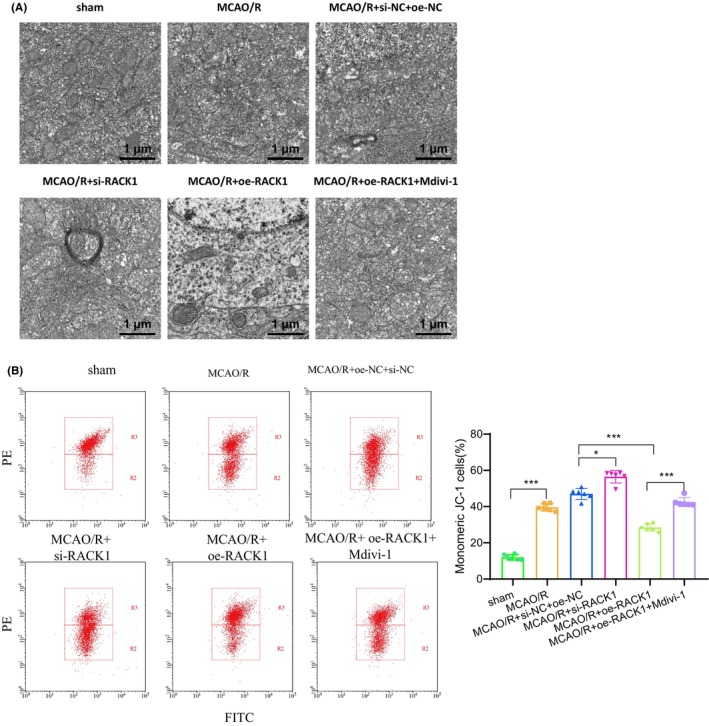
The effect of RACK1 on mitochondrial injury induced by MCAO/R. (A) TEM observation of mitochondrial autophagy in rat brain tissues of different groups (bar = 1 μm); (B) Measurement of mitochondrial membrane potential changes in neurons of rat brain tissues using JC‐1 staining. *p < 0.05. Each group consisted of six rats.

Moreover, we conducted additional investigations into the autophagic state and functionality of neuronal mitochondria in rat brain tissues by employing TEM. Compared to the control group, the MCAO/R group exhibited pronounced damage to mitochondrial structure, vacuolization, and reduced autophagy. Rats with silenced RACK1 exhibited an increase in damaged mitochondria and a decrease in autophagy compared to their control groups. The overexpression of RACK1 in rats leads to a reduction in damaged mitochondria and an increase in autophagy phenomena. However, following the administration of Mdivi‐1, mitochondrial autophagy was notably suppressed, suggesting that Mdivi‐1 negated the stimulatory impact of RACK1 overexpression on mitochondrial autophagy (Figure 7A). Mitochondrial membrane potential was measured in neurons, and the results revealed that the MCAO/R group exhibited a reduced potential compared to the control group, indicating impaired mitochondrial function. Compared to their control groups, the mitochondrial membrane potential is further reduced in rats with silenced RACK1, while it is increased in rats overexpressing RACK1. However, administration of Mdivi‐1 reversed the alterations in mitochondrial membrane potential, providing additional evidence that Mdivi‐1 could mitigate the impact of RACK1 on mitochondrial function (Figure 7B).

In summary, the crucial role of RACK1 in the MCAO/R rat model was further confirmed through in vivo experiments. The overexpression of RACK1 enhances mitophagy, reduces mitochondrial damage, and preserves mitochondrial function, consequently reducing neuronal apoptosis ischemic brain volume and improving neurological functional deficits.

### 3.7 Decoding the Role of RACK1 in mitophagy regulation via the PINK1/Parkin pathway: implications for mitochondrial function and apoptosis

**FIGURE 8:**
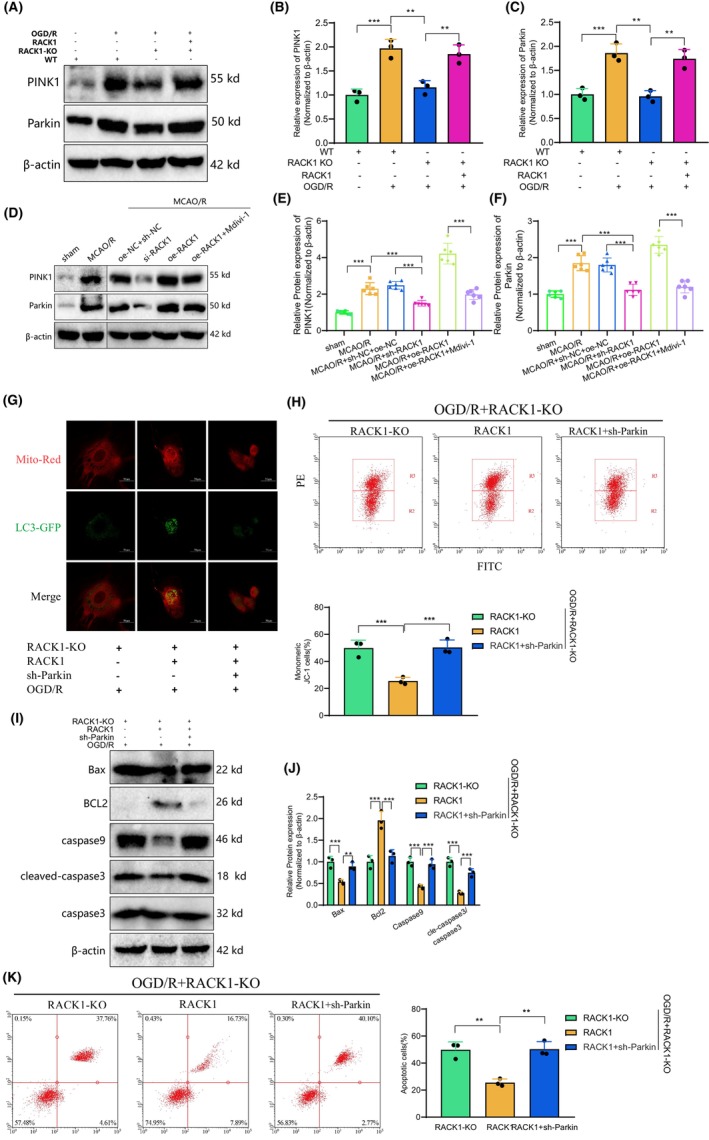
Regulation of mitophagy by RACK1 through the PINK1/Parkin pathway. (A) Protein expression of PINK1 and Parkin detected by Western blot in each cell group; (B, C) Semi‐quantitative statistical analysis of PINK1 and Parkin protein expression; (D) Protein expression of PINK1 and Parkin detected by Western blot in rat brain tissues of each group; (E‐F) Semi‐quantitative statistical analysis of PINK1 and Parkin protein expression; (G) Immunofluorescence staining showing co‐localization of mitochondria and autophagosomes (bar = 10 μm); (H) JC‐1 staining to observe changes in mitochondrial membrane potential in each cell group; (I) Protein expression levels of Bax, Bcl2, caspase‐9, and cleaved‐caspase‐3 detected by Western blot in each cell group; (J) Semi‐quantitative analysis and statistical charts of Bax, Bcl2, caspase‐9, and cleaved‐caspase‐3; (K) Flow cytometry analysis of apoptosis in each cell group. *p* < 0.05, *p < 0.01. Animal experiments were performed using six rats. Cell experiments were repeated three times.

To further investigate the regulatory role of RACK1 in mitochondrial autophagy, we examined the expression of PINK1 and Parkin, which serve as markers for mitochondrial autophagy, in cellular and animal models. The results demonstrated that the expression of PINK1 and Parkin increased following OGD/R treatment. Notably, when RACK1 was knocked out, the expression of PINK1 and Parkin was inhibited, but their expression was restored upon reintroduction of RACK1 (Figure 8A–C). In the rat brain tissues of each group, MCAO/R treatment resulted in an increased expression of PINK1 and Parkin. Conversely, silencing RACK1 led to a decreased expression of PINK1 and Parkin. The overexpression of the RACK1 group showed an increased expression of PINK1 and Parkin, while adding Mdivi‐1 further suppressed their expression (Figure 8D‐F). RACK1 can potentially influence mitophagy by modulating the PINK1/Parkin pathway.

To further investigate whether RACK1's regulation of mitochondrial autophagy depends on the PINK1/Parkin pathway, we conducted a knockdown of Parkin (PRKN) and observed mitochondrial autophagy through immunofluorescence. The results demonstrate that the knockdown of Parkin inhibits the enhancing effect of replenishing RACK1 on mitochondrial autophagy (Figure 8G). By detecting mitochondrial membrane potential, we observed that replenishing RACK1 increased it but suppressed the improvement of membrane potential mediated by RACK1 upon Parkin knockdown. This finding further supports the notion that the modulation of mitochondrial function by RACK1 relies on the induction of mitochondrial autophagy through the PINK1/Parkin pathway (Figure 8H). Moreover, we examined the expression of mitochondrial apoptotic‐related proteins. The results demonstrated that the replenishment of RACK1 suppressed the expression of Bax, caspase 9, and cleaved caspase‐3 while it enhanced the expression of Bcl2. Subsequently, the knockdown of Parkin hindered the impact of RACK1 replenishment (Figure 8I,J). Furthermore, flow cytometry was utilized to validate the findings of cell apoptosis detection (Figure 8K).

In conclusion, it has been observed that regulating mitochondrial autophagy by RACK1 may depend on the PINK1/Parkin pathway. Knocking out RACK1 suppressed the expression of PINK1 and Parkin, while re‐introduction of RACK1 restored their expression levels. Further validation through Parkin knockdown experiments confirmed that RACK1 modulates mitochondrial autophagy through the PINK1/Parkin pathway. Additionally, replenishing RACK1 enhanced mitochondrial membrane potential and inhibited the expression of apoptotic proteins associated with mitochondria, underscoring the significance of RACK1 in regulating mitochondrial function and cell apoptosis.

## 4 DISCUSSION

Cerebrovascular disease has become one of the most common causes of death and disability globally. Post‐acute cerebral ischemia, cerebral ischemia‐reperfusion injury refers to a series of pathophysiological changes occurring after the restoration of cerebral blood flow.^43^ While reperfusion can restore cerebral blood flow, it is also accompanied by adverse effects such as hemorrhagic transformation, disruption of the blood‐brain barrier, and significant edema, which may lead to further neuronal damage.^44^

According to the literature, mitochondria play a significant role in CIRI. Mitochondria serve as the cellular organelles that act as the center for oxidative energy and are essential for cell survival. However, aging or damaged mitochondria serve as a source of toxic ROS. Mitochondrial autophagy is characterized as a selective form of autophagy and plays a crucial role in maintaining cellular homeostasis by clearing damaged and unnecessary mitochondria.^34^ Currently, mitochondrial autophagy has shown a positive role in renal disease, ischemic stroke, and ischemia‐reperfusion injury.^45^

This study presents evidence from both in vitro and in vivo experiments, indicating an upregulation of RACK1 in MCAO/R‐induced cerebral ischemia‐reperfusion. The increase in RACK1 expression underscores its possible role in the pathogenesis of cerebral ischemia‐reperfusion injury (CIRI), which may contribute to the regulation of mitochondrial autophagy and inhibition of neuronal apoptosis through the PINK1/Parkin pathway.^15^, ^46^, ^47^

Our findings illuminate the potential neuroprotective effects of RACK1 by regulating mitochondrial autophagy in CIRI.^48^ These insights align with previous research emphasizing the significant role of mitophagy in various neurological conditions, further supporting its important contribution to maintaining mitochondrial equilibrium and function, which is essential for neural injury recuperation.^49^, ^50^, ^51^, ^52^

By carefully examining the expression and activity of RACK1 during ischemia‐reperfusion, our research suggests a conceivable link between RACK1 and the esteemed PINK1/Parkin pathway. This potential highlights the possible significance of RACK1 as a key regulatory molecule in the intricate dynamics of mitophagy within the realm of CIRI, contributing to our understanding of the comprehensive pathophysiological landscape of CIRI.^53^

Our study contributes to the existing literature on RACK1 by delving into its broader impact on other mitochondrial functions, such as ROS accumulation, mitochondrial membrane potential, and the release of mitochondrial cytochrome C. This approach underscores the importance of targeting mitochondrial functions in developing future therapeutic strategies for better clinical outcomes.

The strength of this research is supported by employing both in vitro and in vivo experimental paradigms, addressing some limitations faced by previous studies focused on singular models. This dual‐model approach enhances the credibility of our findings and adds substantial clinical relevance.

However, it is essential to acknowledge the intrinsic limitations, including the use of specific human neuroblastoma SK‐N‐SH cells and rat models. These models, while applicable, may only partially encapsulate the complexity of brain cell behavior in CIRI across diverse biological contexts. Further investigation employing a broad spectrum of cell types and animal models is essential to refine the understanding of RACK1's role in CIRI and its regulatory influence on the PINK1/Parkin pathway.

In conclusion, this study makes a meaningful contribution to elucidating the intricate molecular mechanisms of CIRI and advances the exploration of innovative therapeutic targets. The identified association between RACK1 and the PINK1/Parkin pathway introduces a novel neuroprotective mechanism, offering a valuable direction for further research and therapeutic advancement. Nonetheless, the journey for in‐depth comprehension and clinical remains ongoing, requiring further comprehensive and detailed research endeavors in this vital biomedical domain.

## AUTHOR CONTRIBUTIONS

**Jinwei Li**: conceptualization, methodology, formal analysis, investigation, writing—original Draft. **Yu Chen**: data curation, software, validation, writing—review and editing. **Hongxi Li**: resources, data curation, supervision, project administration. **Xiaoxu Ding**: visualization, investigation, data curation. **Lanqing Zhao**: conceptualization, resources, supervision, funding acquisition, writing—review and editing, Correspondence. All authors contributed to the revision of the manuscript, read and approved the submitted version.

## FUNDING INFORMATION

This study was supported by the Liaoning Xingliao Talent Program (YXMJ‐JC‐05).

## CONFLICT OF INTEREST STATEMENT

The author declares no conflict of interest.

## Supporting information


Figure S1.

Table S1.

## Data Availability

The data that supports the findings of this study are available on request from the corresponding author.
