# Supplementary material for: Deciphering the neuroprotective mechanisms of RACK1 in cerebral ischemia‐reperfusion injury: Pioneering insights into mitochondrial autophagy and the PINK1/Parkin axis
Source: CNS Neurosci Ther. 2024 Aug 4;30(8):e14836. doi: 10.1111/cns.14836 (PMC11298203; doi:10.1111/cns.14836)
Supplement: Supplementary file 2 — Table S1. [file CNS-30-e14836-s002.docx]

**Table S1. Silent chronic virus sequence**

| Name. | Sequence（5’-3’） |
| --- | --- |
| sh-NC | CCTAAGGTTAAGTCGCCCTCG |
| sh-Parkin-1 | CGCAACAAATAGTCGGAACAT |
| sh-Parkin-2 | CGTGATTTGCTTAGACTGTTT |

**Table S2. RT-qPCR primer sequence**

| Gene | primer sequence |
| --- | --- |
| RACK1 (human) | Forward: 5’- GCTGATGGCCAGACTCTGTT-3’ |
|  | Reverse: 5’- TTCTAGCGTGTGCCAATGGT-3’ |
| RACK1 (rat) | Forward: 5’- GTGCTCTTCGAGGTCACTCC-3’ |
|  | Reverse: 5’- TGTGAGATCCCAGAGGCGTA -3’ |
| Parkin/PRKN（human） | Forward: 5’- GACAGCAGGAAGGACTCACC-3’ |
|  | Reverse: 5’- GCTGCACTGTACCCTGAGTT-3’ |
| GAPDH(human) | Forward: 5’- TGCAACCGGGAAGGAAATGA-3’ |
|  | Reverse: 5’- GCATCACCCGGAGGAGAAAT-3’ |
| GAPDH(rat) | Forward: 5’-GGTACAACTCAGGTTCCGGG -3’ |
|  | Reverse: 5’- ATCCGTTCACACCGACCTTC-3’ |
